# Colonic Cancer and Acromegaly

**DOI:** 10.3389/fendo.2019.00390

**Published:** 2019-06-21

**Authors:** Dorota Dworakowska, Ashley B. Grossman

**Affiliations:** ^1^Department of Hypertension and Diabetes, Medical University of Gdansk, Gdansk, Poland; ^2^Guys Richard Dimbleby Department of Cancer Research, King's College London, London, United Kingdom; ^3^Endocard LTD, London, United Kingdom; ^4^Oxford Centre for Diabetes, Endocrinology and Metabolism, University of Oxford, Oxford, United Kingdom; ^5^Barts and the London School of Medicine, Centre for Endocrinology, William Harvey Institute, London, United Kingdom

**Keywords:** colon, cancer, acromegaly, colonoscopy, screening

## Abstract

Acromegaly results in a significantly increased morbidity and mortality due to cardiovascular and respiratory complications, as well as malignancies arising mainly from the colon. Furthermore, an increased lifetime risk of malignant transformation of pre-malignant colonic lesions relates to a worse overall prognosis from colorectal cancer, which is currently considered a major disease-related complication. In this review we provide some insight into colonic changes in this condition, summarize current knowledge and evidence on the use of colonoscopic screening in patients with acromegaly, and suggest a recommended screening protocol.

## Introduction

Acromegaly is a rare disease with an annual incidence of ~4–6 cases per million per year, with an equal distribution between genders. Epidemiological data suggest that acromegaly is associated with increased morbidity and mortality, mainly from diabetes mellitus, cardiovascular and cerebrovascular disease, as well as from respiratory complications (1–3). However, this increased mortality rate may also include neoplastic processes (2). Nevertheless, while patients with acromegaly have a 2–2.5-fold increased mortality rate predominantly due to non-cancer related reasons, an accurate assessment of the true incidence of cancer in this group of patients remains challenging (2).

Several small studies have shown that patients with acromegaly (compared to the general population) are at a significantly increased risk of developing adenomatous colonic polyps and colorectal cancer (4–10). The largest meta-analysis of colorectal neoplasia in acromegaly was published in 2008 (11). This analysis was based on MEDLINE and EMBASE database searches (up to the end of 2007) and included controlled studies reported in English and published as full-text articles. Only 9 out of 106 initially-generated, potentially eligible studies fulfilled the inclusion criteria. The data collected from 701 acromegalic patients compared to 1,573 controls showed a significantly increased risk of developing colorectal adenomatous polyps, as well as colorectal cancer, in acromegaly in comparison to controls (11). For colonic adenomas the pooled odds ratios (ORs) with 95% confidence limits (CI) were 2.486 (1.908–3.238) (fixed effects model) and 2.537 (1.914–3.364) (random effects model). For colon cancer the pooled OR with 95% CI was identical for both fixed and random effects models (OR = 4.351 with 95%CI 1.533–12.354). These results support the hypothesis of an increased risk of malignant transformation in the colon in acromegaly (11, 12).

These findings have since been confirmed by further case and epidemiological studies, including our own cancer surveillance program (13), involving nearly 700 patients with acromegaly, who had a 2.4-fold increased risk of colonic adenomas and a 7.4-fold greater risk of cancer, with an overall prevalence of colorectal cancer of 3.7% (8, 9, 14, 15).

Furthermore, an association of acromegaly with the development of benign and malignant colonic neoplasms (4, 16) has recently being confirmed in Japanese cohort (17). The diagnosis of acromegaly is usually preceded by growth hormone (GH) hypersecretion for at least 10 years (18). This could not only result in the development of colon polyps, but also possibly allow for premalignant lesions to transform into a cancer (18). Successful control of GH and insulin-like growth factor-1 (IGF1) excess reduces the mortality of these patients to rates seen in the general population, particularly when they are delivered by a multimodal and integrated approach (19).

## Pathophysiology

The pathogenesis of colonic neoplasia in acromegaly is thought to be caused by the increased serum GH and IGF1 levels (20), such excess may facilitate the growth of either pre-existing colonic tumors, or initiate their development (21). GH and IGF1 are known to promote both cellular growth as well as proliferation, and possibly induce proto-oncogene expression (22). IGF1 has been shown to have not only mitogenic but also anti-apoptotic activity (23, 24). Multiorgan neoplastic transformation in rats subjected to excess of GH exposure has been shown in some studies, with the IGF1 receptor being found to be present in the colonic epithelium (20, 25, 26).

The effects of GH are mediated by the GH receptor (GHR). Binding of GH to the GHR activates signal transduction pathways critical for cell growth and survival, including the Janus kinase-2/signal transducers and activators of transcription (JAK-2/STAT), c-Src (p44/42 mitogen activated protein kinase (MAPK), and the phosphoinositide 3-kinase (PI3K) pathways. Upregulation of components of these pathways has been observed in a wide range of malignancies (27). GH also induces early response genes that activate cell growth and differentiation signals mediated by CCAT enhancer-binding protein-b and serum response element sites on the c-fos promoter (28). GH/IGF1 was shown to have anti-apoptotic properties in several cell lines including human colonic adenocarcinomas (26, 29), however, the underlying molecular mechanisms seem to be different in specific types of cell lines. For example, increased apoptosis has been shown in cardiac myocytes of patients with acromegalic cardiomyopathy, and in myocytes of patients with critical illness treated with GH (30). Interestingly, in patients with acromegaly, increased colonic epithelial cell proliferation was seen (26) in addition to reduced apoptosis in colonic mucosa (29). Cats et al. (26) reported that patients with acromegaly had an increased proliferation index of colonic epithelium proportional to their circulating IGF1 levels. Increased circulating IGFBP3, IGFBP2, and IGF2 levels may also play an important role (31). Thus, it is currently considered that the biological basis for the association between colonic neoplasms and acromegaly results from the known mitogenic actions of GH/IGF1, together with slow intestinal transit in in a usually enlarged and redundant large bowel (24, 26, 32).

The effects of GH on somatic growth appear to occur principally by induction of hepatic IGF1 secretion. The tumorigenic process is modulated by the IGF1 system at different levels. In experimental studies, knockout of the IGF1-R gene decreased cell proliferation and increased apoptosis (33). The IGF1/IGF1R system may also influence cancer progression due to promoting adhesion and migration of cells, as well as angiogenesis within tumor tissues and in the surrounding areas (34). There is expression of the IGF1R mRNA in colon cancer tissue, and this may be involved in paracrine and autocrine effects as well as circulating IGF1 (33).

Recent research has highlighted the involvement of not only IGF1 signaling but also GH signaling itself in colon tumorigenesis. Signal transducer and activator of transcription-5 (STAT5) protein (known to be activated by GH) has been shown to play an important role in tumor progression through the stimulation of cell proliferation and the prevention of apoptosis. STAT5 activation is involved in the development of prostate cancer, breast cancer and leukemia (35–37). Colorectal adenocarcinomas show higher levels of STAT5b expression than normal colonic mucosa, and the expression levels are associated with the TNM stage (38). STAT5 phosphorylation has often been observed in colon adenocarcinomas and is associated with a poor prognosis (39). Thus, direct activation of Stat5 by GH may be oncogenic, independent of IGF1.

## Risk factors for colon cancer

**Figure 1:**
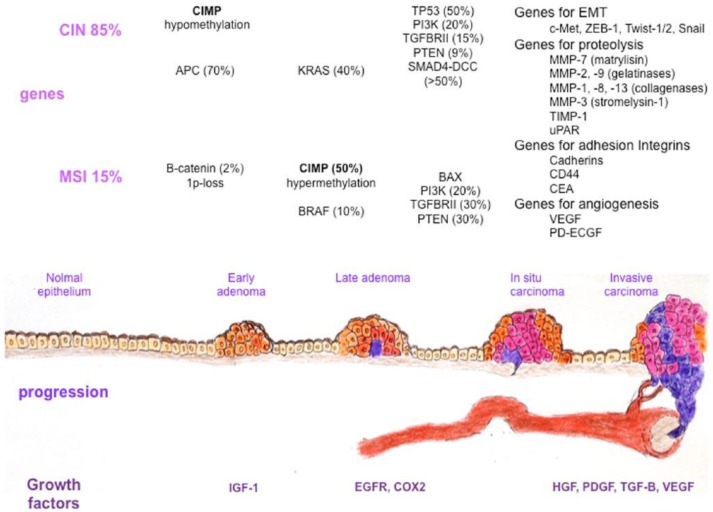
Colorectal cancer heterogeneity during the adenoma-carcinoma sequence. Several factors, such as growth factors and genetic or epigenetic alterations in specific oncogenes or tumor suppressor genes, confer a selective advantage to epithelial cells for proliferation and self-renewal. The normal epithelium becomes a hyperproliferative mucosa and subsequently gives rise to a benign adenoma, which can then evolve into a carcinoma and metastases. Different genetic changes characterize colorectal cancer progression in tumors with microsatellite instability and chromosomal instability. Alterations in genes involved in epithelial to mesenchymal transition, basal membrane degradation, cell adhesion, and angiogenesis are finally responsible for metastasis development. CIN, chromosomal instability; CIMP, CpG island methylator phenotype; MSI, microsatellite instability; APC, adenomatous polyposis coli; KRAS, Kirsten Rat Sarcoma viral oncogene homolog; TP53, Tumor Protein 53; PI3K, Phosphoinositide 3, Kinase; TGFBRII, Transforming Growth Factor Beta Receptor II; PTEN, Phosphatase and Tensin homolog; SMAD4-DCC, Small Mother Against Decapentaplegic 4-Deleted in Colorectal Carcinoma; c-MET, Mesenchymal to Epithelial Transition factor; ZEB-1, Zinc finger E-box Binding homeobox-1; MMP, Matrix Metalloproteinase; TIMP-1, Tissue Inhibitor of Metalloproteinase-1; uPAR, urokinase-type Plasminogen Activator Receptor; CD44, Cluster of Differentiation 44; CEA, Carcinoembryonic Antigen; VEGF, Vascular Endothelial Growth Factor; PD-ECGF, Platelet Derived-Endothelial Cell Growth Factor; B-RAF, v-raf murine sarcoma viral oncogene homolog B1; BAX, Bcl2-Associated X protein; IGF-1, Insulin Growth Factor-1; EGFR, Epidermal Growth Factor Receptor; COX2, Cyclooxigenase 2; HGF, Hepatocyte Growth Factor; PDGF, Platelet Derived Growth Factor; TGF-B, Transforming Growth Factor Beta; VEGF, Vascular Endothelial Growth Factor [presented by De Rosa et al. (40)].

The majority of colon cancers develop as a result of the multistep malignant transformation of benign adenomatous colonic polyps: this takes ~10–15 years in non-acromegalic individuals (18). Colon cancer usually develops or evolves from pre-malignant adenomatous lesions (Figure 1) (18, 40, 41). It has been variably associated with a wide range of predisposing factors including diet, obesity, diabetes, and smoking, as well as genetic and epigenetic mechanisms (Table 1) (42–46).

**Table 1:** Colon cancer risk factors in general population.

**Modifiable**	**Non-modifiable**
Diet (high in red meats, processed meats, low in fiber, high in fat) DM2 Physical activity Obesity Smoking Alcohol use	Age Personal history of inflammatory intestinal conditions Family history Race

Obesity, diabetes, hypertriglyceridemia and physical inactivity (associated with hyperinsulinaemia) are conditions with an increased prevalence of colorectal tumors in the general population (47, 48), leading to the hypothesis that hyperinsulinaemia may play a role in the colorectal tumorigenesis (49).

Potential risk factors for the induction of colorectal cancer in acromegaly have been investigated in detail. These include hyperinsulinaemia, but altered acid bile salt secretion, changed local immune responses, increased large bowel length and altered bowel transit times could also contribute to an adenoma occurrence/recurrence in patients with acromegaly [reviewed by Lois et al. (12)]. Patients with acromegaly have an increased prevalence of insulin resistance leading to hyperinsulinaemia (33). Two studies have reported an association between high insulin levels and an increased risk of colonic lesions in acromegalic patients (50, 51), although this was not supported by another study (49). However, type 2 diabetes has been consistently associated with an increased risk for colorectal carcinoma (52, 53). Moreover, while such risk appears to be further increased in patients who use insulin, treatment with metformin and thiazolinedieniones has been found to reduce it substantially (43, 44). Although abnormalities of glucose metabolism are highly prevalent in acromegaly, some studies have reported no difference in the frequency of diabetes between patients with and without colonic lesions (54, 55).

Diabetes mellitus seemed to be more frequent among patients with colonic polyps in the study by Gonzalez et al., although their multivariate analysis did not identify diabetes as an independent risk factor for the development of these colonic lesions in patients with acromegaly (31). In that study the main objectives were to establish the real prevalence of colonic polyps in patients with newly-diagnosed active acromegaly (in comparison to 949 healthy adult individuals undergoing colonoscopy screening as part of a medical check-up), and to identify clinical and biochemical features that would distinguish patients with and without such colonic lesions. They found no correlation between age, gender, diagnosis delay, or the prevalence of hypertension or dyslipidemia, and the presence of colonic polyps in patients; as expected, diabetes mellitus was significantly more frequent among patients with acromegaly than in controls (31).

Obesity is a consistent risk factor for the development of colonic cancer in the general population (43, 45). The link between a high BMI and colon cancer was seen statistically robustly in men with central obesity (43, 45). Patients with acromegaly usually have an increased BMI related to an increased lean body mass, yet the majority of studies have not found patients with colonic polyps or cancer to be more frequently obese or overweight than those without colonic lesions (31, 54, 55).

The common C677T polymorphism in methylenetetrahydrofolate reductase (MTHFR) gene is a known risk factor for colorectal tumors (CRT) in the general population (56). By contrast, high serum 25-hydroxy vitamin D3 [25(OH)D_3_] concentrations have been related to a *decreased* risk of colorectal cancer and adenomas in the general population (57, 58), with vitamin D supplementation appearing to reduce the risk of developing colonic cancer (59). Indeed, some studies in the general population showed that a high folate intake may reduce the risk of colorectal cancer and adenomas (60, 61). Further studies have attempted to address the same factors in acromegaly. Torre et al. investigated the influence of the MTHFR C677T polymorphism, folate status and lifestyle factors on CRT risk. Clinical data were collected from 115 patients with acromegaly (25 with active disease) who underwent a complete colonoscopy. C677T MTHFR genotype, homocysteine, vitamin B_12_, IGF1 and insulin levels, as well as metabolic variables were evaluated. They found that CRT risk was increased in 677TT MTHFR patients with low plasma folate levels. Smoking, high HbA_1C_ levels, dyslipidemia and disease activity were also associated with increased CRT risk (56). Lombardi et al. analyzed a cohort of 146 consecutive patients with acromegaly, and a pilot study was conducted in 9 naive acromegalic patients to evaluate the effect of somatostatin analogs (SSA) on serum levels of those factors. Levels of insulin were reduced during SSA therapy while the other factors did not change. In that cohort study, colonic lesions (14 adenomas; 32 hyperplastic polyps) were detected in 46 patients, but fasting insulin, 25(OH)D3, folate, and homocysteine levels did not differ in patients with or without colonic adenomas. High folate levels were associated with a lower risk of developing precancerous colonic lesions on multivariate analysis, when corrected by age, gender, disease activity and SSA therapy. Lombardi et al. therefore concluded that serum insulin, 25(OH)D3 and homocysteine serum concentrations probably do not influence the development of precancerous colonic lesions in patients with acromegaly, while higher folate levels may be associated with a lower risk of colonic lesions and thus folate may have a protective role in the development of colonic neoplasms (49).

## Risk of Colorectal Cancer-Specific Mortality

The question as to whether the increased risk of colorectal cancers in acromegaly results in increased colorectal cancer-specific mortality in this group remains unanswered (12). This topic has been summarized by Lois et al., concluding that while though the initial studies suggested increased overall cancer related mortality in acromegaly, further studies have failed to support these findings (4, 14, 62, 63), even though patients with acromegaly had been reported to have a nearly a 2.5-fold higher colon cancer specific mortality rate compared to the general population (14). In one large study, the mortality rate due to colon cancer was found to be increased in relation to high GH levels (14), but disease activity seemed not to play any major role in patients who had no colorectal lesions at their first colonoscopy (64) [although it is worth to note that in a recent case report a patient died from colon cancer with negative initial colonoscopy (12)]. Furthermore, in a large meta-analysis Rokkas et al. concluded that an overall cancer mortality risk was significantly greater in a subgroup of patients with uncontrolled GH levels (11). However, as many centers now undertake routine colonoscopy (see below), perhaps these findings are less surprising.

## Colonoscopy Screening Guidelines in the Light of a Real-Life Practice

As discussed above, patients with acromegaly have an increased prevalence of both pre-cancerous and malignant colonic lesion in comparison to the general population. Therefore, it is generally advised that patients with acromegaly should have colonoscopic screening performed more frequently than in the general population (12). In guidelines published in 2002, Melmed et al. recommended that colonoscopy should be performed every 3–5 years depending upon clinical indications, including assessment of family history and previous polyp detection (65). In their update in 2009, these authors suggested that at least one baseline colonoscopy assessment is required in all patients with acromegaly, and that patients with colonic polyps should be followed according to the international guidelines for colon cancer (66–68). Even though multiple authors have suggested guidelines for routine screening and surveillance colonoscopy in acromegaly patients, this practice has not been widely followed (22, 69, 70).

Our own findings strongly supported an evidence base for a regular surveillance programme in all patients with acromegaly, irrespective of the findings on the initial colonoscopy (13, 22). We have shown that patients with an initial adenoma had a 4.4- and 8.8-fold increased risk of developing a new adenoma at the second and the third colonoscopy, respectively, while patients with a normal initial colonoscopy and persistently elevated IGF1 levels had 7.5-fold risk of a subsequent adenoma, compared to those with a normal colonoscopy at the initial screening and inactive disease (13). In spite of the uncertainties as to whether uncontrolled acromegaly is associated with an increased death rate from colorectal cancer (as noted above) (54, 71, 72), international consensus guidelines still recommend more frequent colorectal cancer screening, especially in patients with uncontrolled IGF1 levels.

In 2010, The British Society of Gastroenterology (BSG) and the Association of Coloproctology for Great Britain and Ireland (ACPGBI) commissioned an update of the 2002 guidance. The authors provided guidance for gastroenterologists on the appropriateness, method and frequency of screening for people at moderate and high risk from colorectal cancer, including colonoscopy screening in acromegaly (73). They suggested that patients with acromegaly should be offered regular colonoscopic screening, starting at the age of 40 years (Recommendation grade: B), and that the frequency of repeat colonoscopy should depend on the findings at the original screening and the activity of the underlying acromegaly (Recommendation grade: B). In particular, patients with an adenoma at first screening *or* an elevated serum IGF1 level above the maximum of the age-corrected normal range should be offered a screening every 3 years. Patients with a negative first colonoscopy or a hyperplastic polyp, or normal growth hormone/IGF1 level, should be offered screening every 5 to 10 years (73). The majority of prospective series have shown a positive association between the prevalence of adenomas and increasing age, although a recent large series reported significantly increased prevalence in patients under 40 years of age compared with a control group (19% vs. 4.4%) (9).

In order to obtain preliminary data on appropriate screening, two groups have performed repeat colonoscopy on their original cohort of patients (9, 22). New adenomas were found in 14–15% of the cohort overall, but in 25–41% of those who had an adenoma at the original screening (at a mean interval of approximately 32 months after the original screening colonoscopy). Interestingly, >90% of patients who developed new adenomas had either neoplasia at the original colonoscopy or an elevated serum IGF1. Therefore, significant risk factors included a presence of an adenoma on initial screening but also elevated growth hormone or serum IGF-1 levels, i.e., uncontrolled acromegaly (9, 22). In addition, these studies recommended a pan-colonoscopy due to the fact that 25–40% of adenomas and 50% of carcinomas occurred in the ascending or transverse colon (8, 9).

To provide a balanced view on the topic, we should emphasize that some authors feel that there are still insufficient data to advocate an intensive colorectal cancer screening programme for patients with acromegaly. Perry et al. note that the increased risk of colorectal cancer is modest and the potential risk of colonoscopy in acromegalic patients is considerable. They suggested that the guidelines need to be challenged before gastroenterologists are forced into a practice which is in their view not evidence-based and may potentially be detrimental to patient well-being (70). However, our own opinion is that the risks of colonoscopy are minor, even in patients with acromegaly who need more intensive preparation and examination, and these are outweighed by the benefits of the early diagnosis of cancer, even this is demonstrable in just a minority of patients.

## Practical Issues Influencing Colonoscopy Success (74)

In acromegaly a total pan-colonoscopy is required rather than sigmoidoscopy or limited colonoscopy. This is based on the studies showing that, as opposed to the general population, in acromegaly 25% of adenomas and 50% of carcinomas occur in the ascending and transverse colon (8, 11, 54, 75, 76). Cairns et al. confirmed that a total colonoscopy is required rather than sigmoidoscopy, although the former may be associated with technical difficulties (73).

In acromegaly, several issues affect the success of total colonoscopy, including increased length of colon (mainly the sigmoid section) and increased circumference (8, 77). Furthermore, colonic transit times are twice longer than in normal individuals, and therefore standard bowel preparation is often inadequate (73). Some authors suggest that twice the “standard” preparation of polyethylene glycol electrolyte solution gives a better chance for a good result: however, individual patients may require even more preparation. The duration of the procedure remains much longer due to the colonic length and circumference. Therefore, the study needs to be done by an experienced operator with adequate skills (8, 73, 74, 78).

In summary, we agree that the technical aspects of colonoscopy in acromegaly require further studies to obtain evidence as to how to optimally prepare the patients for this examination.

## FECAL Occult Blood

Gogazzi et al. compared colonoscopy and fecal occult blood testing (FOBT) as a first-line screening of colonic lesions in patients with newly-diagnosed acromegaly (79). In that study, colonoscopy and FOBT were performed at the first diagnosis of acromegaly in 85 consecutive patients with untreated active acromegaly. FOBT was positive in 16 (18.8%) out of 85 patients and identified 2 patients with colonic adenocarcinoma and 2 with adenomas; the remaining 12 patients had no detectable colonic lesions. Colonoscopy revealed colonic lesions in 29 patients: 3 (3.5%) cancers, 11 (12.9%) adenomas, and 15 (17.6%) hyperplastic polyps. The remaining 56 patients with acromegaly had no detectable lesions. In the light of the fact that a patient with cancer and 9 patients with adenomas were missed if screened only by FOBT, the authors concluded that colonoscopy was superior to FOBT in detecting colonic lesions at the first diagnosis of acromegaly (79).

## Computed Tomographic (CT) Colonography (“Virtual Colonoscopy”)

A systematic review and meta-analysis by Pickhardt et al. assessed the sensitivity of both computed tomographic (CT) colonography and optical colonoscopy (OC) for colorectal cancer detection in the general population, reporting that primary CT colonography may be more suitable than OC for the initial investigation of suspected colorectal cancer (80). CT tomographic colonography is an innovative and secure technology which may revolutionize the diagnosis of colon/rectum neoplasms. Ramos et al. analyzed its performance of this technology for colorectal polyp screening in a prospective study of patients with acromegaly (21 asymptomatic acromegalic patients, 12 male and 9 female, average age 49), who underwent CT colonography and conventional colonoscopy. CT colonography was complete in all cases; however, in 2/21 patients, conventional colonoscopy was incomplete. In Phase I ('per patient'), CT colonography diagnosed 8/9 patients with colorectal polyps (88% sensitivity, 75% specificity, 81% accuracy). In Phase II (“per polyp”), of the 21 acromegalic patients included in this study, 12 presented normal findings at conventional colonoscopy: 10/19 polyps detected in 9 patients were <10 mm, and 9 were ≥10 mm. CT colonography identified 7/9 polyps >10 mm described by conventional colonoscopy, but only 6/10 small polyps identified at conventional colonoscopy were detected by CT colonography. The group concluded that CT colonography was performed without complications and that a complete and safe colorectal evaluation was possible in all patients with acromegaly with acceptable sensitivity, specificity and accuracy for the identification of polyps of any size. However, this method gave better results in the diagnosis of large polyps when they were compared to small polypoid lesions (81, 82). Resmini et al. also assessed the feasibility and results of CT colonography in acromegaly. They examined 23 patients with acromegaly with no history of colorectal cancer (11 females and 12 males; age range 18–79 years; disease duration range 1–15 year). They found that CT colonographic examination results were adequate in 17 of 23 cases (73%) and demonstrated 12 polyps in 8 patients with 95% being confirmed by traditional colonoscopy. Importantly, there were no polyps found by traditional colonoscopy that CT colonography was not able to identify. The authors concluded that CT colonography has the potential ability to replace traditional colonoscopy in this patient group (81, 82).

It is worth mentioning that CT colonography, as a non-invasive study, does not allow for intervention and is clearly associated with increased radiation, which may not be acceptable for a screening programme. However, some patients with acromegaly have a colon which is often too difficult for complete intubation or have co-morbidities which significantly increase the risk of adverse events. For those patients (similar to general population in such cases), CT colonography is a safe alternative with excellent diagnostic performance (83).

## Costs and Benefits

The small number of cases of acromegaly in the UK means that assessment of the cost-benefit ratio is very difficult to gauge. There are ~2,500 patients with acromegaly in the UK, of whom about 2,000 are aged 40 years or over. About 25% of these (500), according to current data, will have an adenoma and thus would be offered 3-yearly screening, while the remainder would be offered screening every 5–10 years. Thus, the number of extra examinations in each center due to acromegaly is likely to be small [summarized by Cairns et al. (73)].

## Acromegaly and Other Colonic Conditions

As noted and discussed above, it is now generally accepted that the prevalence of colon polyposis/malignancies is increased in patients with acromegaly. An epidemiological study also suggests a high prevalence of small bowel (SB) tumors nowadays detectable by videocapsule endoscopy (VCE). Ronchi et al. assessed the prevalence of SB neoplasms using VCE in patients with acromegaly in comparison to control subjects and correlated it with cancer risk factors and acromegaly-related parameters. The study group included 18 acromegalic patients (6 males and 12 females, 54 ± 10 years old) (5 cured after surgery, followed by radiotherapy in 3 cases and 13 on pharmacological treatment). VCE images suggestive of SB lesions were detected in 5/36 controls [14%, 4 described as gastrointestinal stromal tumors (GIST), and 1 as a polyp] and in 5/18 patients with acromegaly [28%, 2 GIST and 3 polyps]. In acromegaly, the calculated relative risk for all SB lesions was 1.69 (95% CI 0.78–3.65), while the relative risk for SB polyps was 2.50 (95% CI 1.23–5.07). The effective duration of active disease was longer in patients with positive than in those with negative VCE (112 ± 89 vs. 49 ± 40 months, *p* = 0.06). These preliminary results suggest that these patients might have a high risk of SB polyp development. VCE might be a useful adjunctive diagnostic tool in acromegaly; however, these findings require confirmation (73).

In a recent case-control study, the authors tried to assess the prevalence of colonic diverticula in patients with cured acromegaly. They screened reports of screening colonoscopies carried out in 107 patients with cured or biochemically-controlled acromegaly compared to 214 age- and sex-matched controls for the presence of diverticula, dolichocolon, and polyps. The findings were subsequently correlated with GH/IGF-I concentrations at the time of diagnosis of acromegaly, and to the duration of GH/IGF-I excess. They found that, in acromegaly, colonic diverticula were present in 37% of patients, dolichocolon in 34%, and adenomatous polyps in 34%, which was significantly increased compared with controls (OR 3.6, 95% CI 1.4–5.7; OR 12.4, 95% CI 6.8–18.0; OR 4.1, 95% CI 1.9–6.4, respectively). The presence of colonic diverticula was associated with increased GH and IGF-I levels at the time of diagnosis. The presence of dolichocolon and adenomatous polyps was associated with higher IGF-I concentrations at diagnosis. This study indicated that not only is acromegaly associated with an increased prevalence of colonic diverticula, but also confirmed an irreversible effect of GH and/or IGF-I on the collagen in the colon (84).

Another study on the gastrointestinal system focused on assessment of the frequency of irritable bowel syndrome (IBS), as it was suspected that in acromegaly the increased bowel length and delayed transit time may cause functional disturbances of the bowel. They included 23 active acromegaly cases and the control group consisted of 90 gender and age-matched healthy individuals. IBS was present only in 1 of 23 of the patients with acromegaly compared to 3/90 controls (*p* = 0.81), and thus that although acromegaly and IBS may cause similar gastrointestinal symptoms, acromegaly is not associated with IBS (85).

## Conclusions

In this review we have focused on the role of colon screening in preventing major acromegaly-related complications. We have discussed disease pathophysiology especially in the context of its influence on colonic function and neoplasia. The reported prevalence of colonic polyps in patients with acromegaly has ranged from 6 to 30% for both adenomatous and non-adenomatous lesions, whereas that of colorectal carcinoma has varied between 4 and 10% (8, 9, 50, 54, 75, 76). According to a large meta-analysis it is estimated that patients with acromegaly are ~2–5 times more likely to develop adenomatous and hyperplastic polyps than non-acromegalic control subjects (11). Controversy regarding the increased risk of colonic neoplasia in acromegaly relates to significant heterogeneity in study design and lack of an ideal control group among the published studies (11).

Current recommendations are based on a variety of sources: the Acromegaly Consensus Group (ACG) guidelines in 2009 (54), our own “Barts” paper in 2010 (13), the British Society of Gastroenterology (BSG) in 2010 (73), American Association of Clinical Endocrinologists (AACE) in 2011 (86, 87) and the Pituitary Society in 2013 (88). In summary, baseline colonoscopy should be done at the time of diagnosis of acromegaly in all adult patients (ACG, AACE), with surveillance commencing at the age of 40 years (Barts, BSG). If a patient has normal findings on initial colonoscopy and normal IGF1 levels, further colonoscopies should be performed every 5–10 years (Barts, ACG, AACE). If baseline/subsequent surveillance colonoscopy reveals the presence of an adenoma, 3-yearly (BSG) or 3–5-yearly (ACG) or 5-yearly (Barts, AACE) surveillance should be recommended. Persistently raised IGF1 levels, regardless of the initial colonoscopy findings, necessitate a repeat colonoscopy every 3–5years (Barts, ACG, BSG) [summarized by Lois et al. (12)]. Specific colonoscopic screening in acromegaly can be abandoned if there is both a normal colonoscopy and the criteria for control of the acromegaly are met (Barts). One would anticipate that with such surveillance screening, the incidence of CRC in acromegaly may well fall below that in the general population.

## Author Contributions

All authors listed have made a substantial, direct and intellectual contribution to the work, and approved it for publication.

### Conflict of Interest Statement

The authors declare that the research was conducted in the absence of any commercial or financial relationships that could be construed as a potential conflict of interest.
